# The complete mitogenome of the entomopathogenic fungus *Metarhizium pinghaense* 15R

**DOI:** 10.1080/23802359.2023.2292145

**Published:** 2023-12-18

**Authors:** Tae Young Shin, Seulki Kim, InJi Heo, Soo Dong Woo, Woo Jin Kim

**Affiliations:** a Department of Agricultural Biology, College of Agriculture & Life Sciences, Jeonbuk National University, Jeonju, Korea; b Department of Agricultural Biology, College of Agriculture, Life & Environment Science, Chungbuk National University, Cheongju, Korea; c EntoCode Co, Seoul, Korea

**Keywords:** Biopesticides, fungal entomopathogens, aphid pathogenic fungus, mitogenome, clavicipitaceae

## Abstract

In this study, the complete mitogenome of the entomopathogenic fungus *Metarhizium pinghaense* 15 R, which is highly virulent to aphids and was isolated from Korean soil, was assembled and annotated for three ATP synthase subunits (*atp*6, *atp*8, and *atp*9), three cytochrome oxidase subunits (*cox*1, *cox2*, and *cox*3), apocytochrome b (*cob*), seven subunits of NADH dehydrogenase (*nad*1*, nad*2*, nad*3*, nad*4*, nad*4L*, nad*5, and *nad*6), two ribosomal RNAs (*rnl* and *rns*), and 19 tRNA genes. Five genes were carrying a total of eight introns, and they may encode ribosomal protein S3, LAGLIDADG and GIY-YIG endonucleases. Phylogenetic analysis based on the mitochondrial nucleotide sequence confirmed that the *M. pinghaense* 15 R is a member of the Clavicipitaceae, and is closely related to the species *M. anisopliae, M. robertsii,* and *M. brunneum.* The mtDNA base sequence of the *M. pinghaense* 15 R strain reported in this study is thought to be useful for biological resource genetic data.

## Introduction

*Metarhizium pinghaense* Q.T. Chen & H.L. Guo, 2020 is a fungus that causes disease in insects and belongs to the family Clavicipitaceae (Ascomycota: Hypocreales). The fungal genus *Metarhizium*, characterized by the formation of green conidia after killing a host insect, was first described by Metschnikoff in 1879 (Zimmermann et al. 1995), and has been isolated from various soils around the world, making it one of the most famous entomopathogenic fungi to date (Shin et al. 2013). Among species belonging to the genus *Metarhizium*, the fungus belongs to the most recently differentiated group, with a wide host range from endopterygota to exopterygota of the insect class, and even spider class (Mongkolsamrit et al. 2020). The genus *Metarhizim* including this species is recognized as an important entomopathogenic fungal group for coevolution studies with insects through genome analysis (Hu et al. 2014). As a result of these interests, the whole genome and mitochondrial sequence of this fungal species have already been determined and used in various studies (Ghikas et al. 2006; Gao et al. 2011). To our knowledge, the complete mitochondrial sequence of *M. brunnem*, *M. anisoplie*, *M. album*, and *M. rileyi* was released in NCBI database (National Center for Biotechnology Information, https://www.ncbi.nlm.nih.gov/). However, the complete mitochondrial sequence of *M. pinghaense* is still unreported. In addition, to secure the population genetics and biological sovereignty for use as microbial pesticides of this worldwide fungal species, it is necessary to secure the mitochondrial sequence of the fungal species isolated from various regions. Therefore, in this study, we present the complete mitogenome of the *M. pinghaense* 15 R strain isolated in Korea, which shows high aphid-insecticidal activity (Heo et al. 2023). The *M. pinghaense* 15 R strain is molecularly characterized by the findings of this study, which can also be used to classify and investigate the evolution of the entomopathogenic fungi *Metarhizuum*.

## Materials and methods

**Figure 1.**
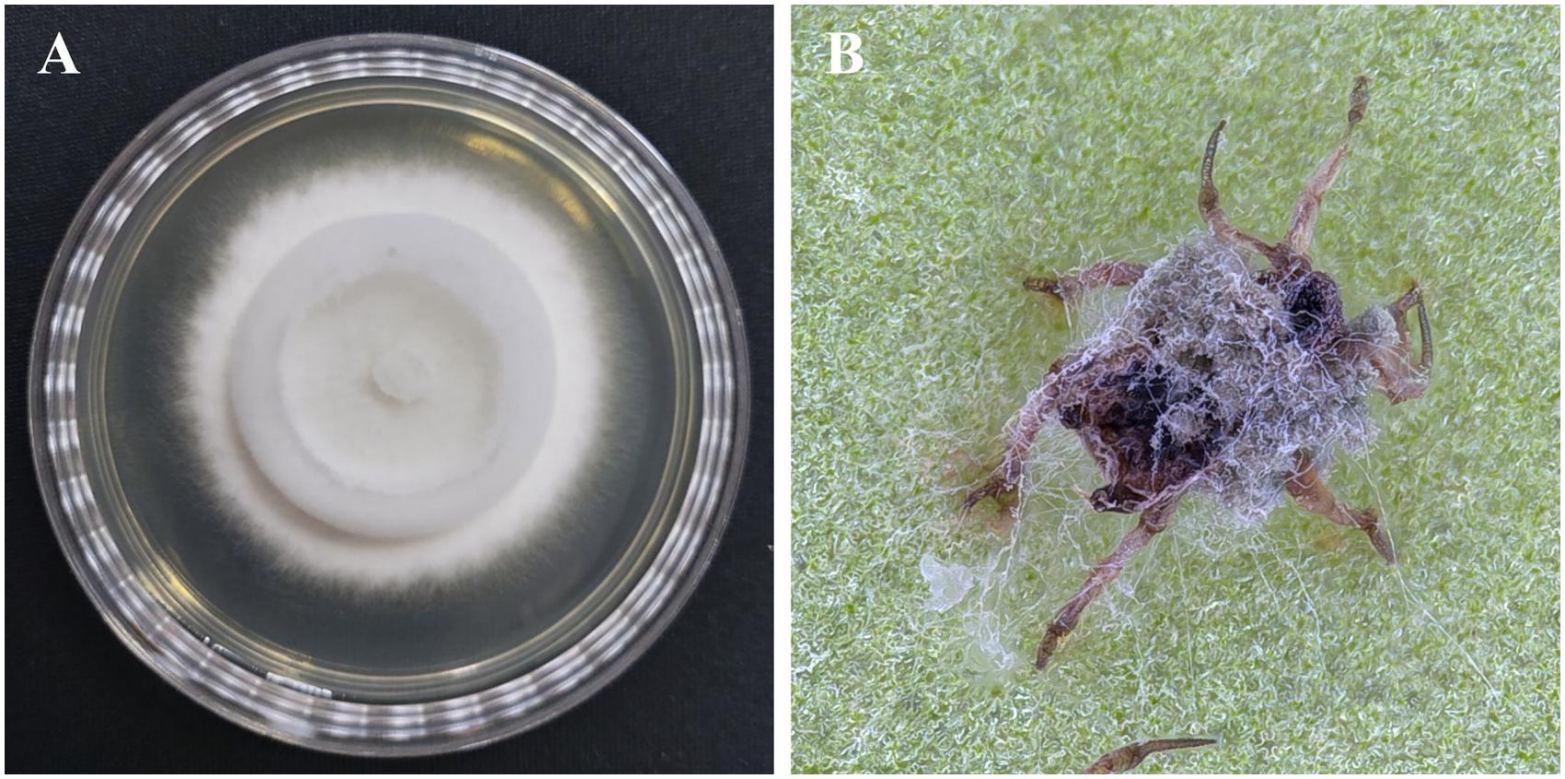
Morphological observation of the *Metarhizium pinghaense* 15 R strain. (A), the strain was cultured on PDA medium at *25 °C* for 2 weeks; (B), green peach aphid (*Myzus persicae*) infected with the strain. The cadaver was covered with the strain’s white hyphae and green conidia and was photographed by InJi Heo at the insect pathology and bioactives laboratory of jeonbuk National university, Korea, on 19 jan 2023.

The *M. pinghaense* 15 R strain was isolated from a Korean soil sample (36°37'43.2"N 127°27'08.1"E), and deposited in the Korean Agricultural Culture Collection (http://genebank.rda.go.kr/, ByeongHak Han, ybyung2000@korea.kr ) under the voucher number KACC 83065BP (Figure 1). This strain shows strong pathogenicity against aphids and is receiving positive attention in research on the development of microbial pesticides.

The total DNA of *M. pinghaense* 15 R was fragmented by sonication to a size of ∼280 bp, followed by sequencing on an Illumina NovaSeq platform in 2 × 150 bp reads. The high quality PE reads were assembled by CLC assembly cell (ver. 4.010.83648, CLC QIAGEN) and Mitoz (Meng et al. 2019), followed by manual curation through PE reads mapping (Kim et al. 2015). Annotation of the complete mitochondrial genome was performed with Geseq (Tillich et al. 2017) and manual corrections (Meng et al. 2019). The coverage map was visualized with the tablet program (Milne et al. 2010).

For the phylogenetic relationship of *Metarhizium pinghaense* 15 R strain, the phylogenetic tree was constructed based on the complete genomes of 11 species with *Verticillium dahliae* NC_008248.1 as the outgroup. Multiple sequence alignments were performed using the MAFFT 7.520 program (Rozewicki et al. 2019), and phylogenetic analysis was performed Maximum likelihood (ML) method of RAxML 8.2.12 (Stamatakis 2014) using the nucleotide substitution model GTR + G + I model with 1,000 bootstraps replicates. The following sequences with GenBank accession were used: *M. album* MW448543.1 (Sun et al. 2021)*, M. anisopliae* NC_008068.1 (Ghikas et al. 2006)*, M. brunneum* LR792747.1 (Kortsinoglou et al. 2020), *M. rileyi* MT107156.1 (Zhang et al. 2020a)*, M. robertsii* JELW01000367, *Akanthomyces lecanii* MN904747.1 (Zhang et al. 2020b)*, Clonostachys rosea* NC_036667.1, *Fusarium solani* NC_016680.1 (Al-Reedy et al. 2012)*, Gibberella moniliformis* NC_016687.1 (Al-Reedy et al. 2012)*, Hirsutella thompsonii* MH367296.1 (Wang et al. 2018)*, Metacordyceps chlamydosporia* NC_022835.1 (Lin et al. 2015), and *Verticillium dahliae* NC_008248.1 (Pantou et al. 2006).

## Results

**Figure 2.**
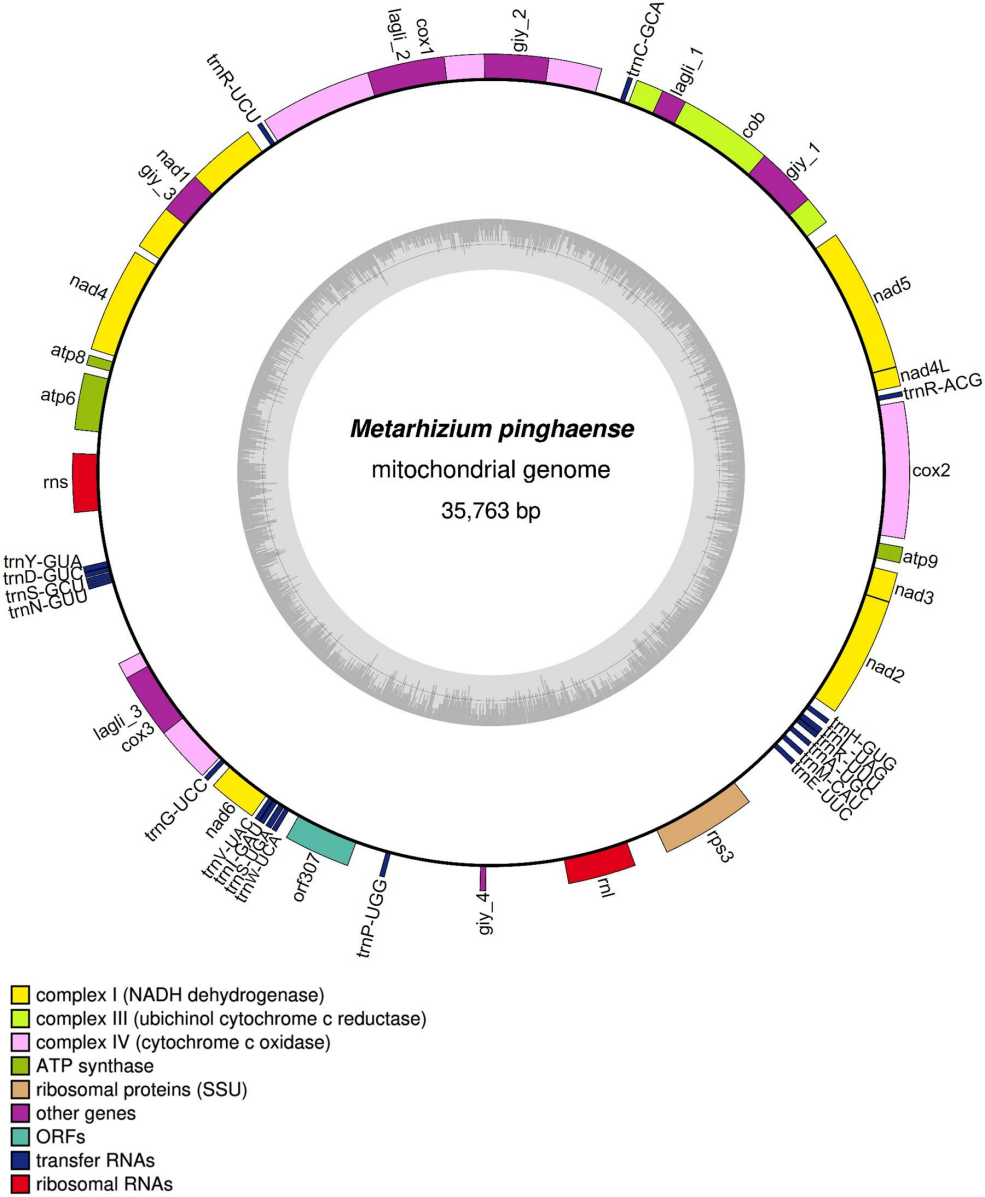
Graphic representation of19 features identified the *Metarhizium pinghaense* 15 R mitochondrial genome. The map was prepared using OGDRAW program (https://chlorobox.mpimp-golm.mpg.de/OGDraw.html). Genes are shown outside, and GC and at contents across the mitochondrial genome are shown with dark and light shading, respectively, inside the inner circle. Genes are color-coded by their functional classification.

**Figure 3.**
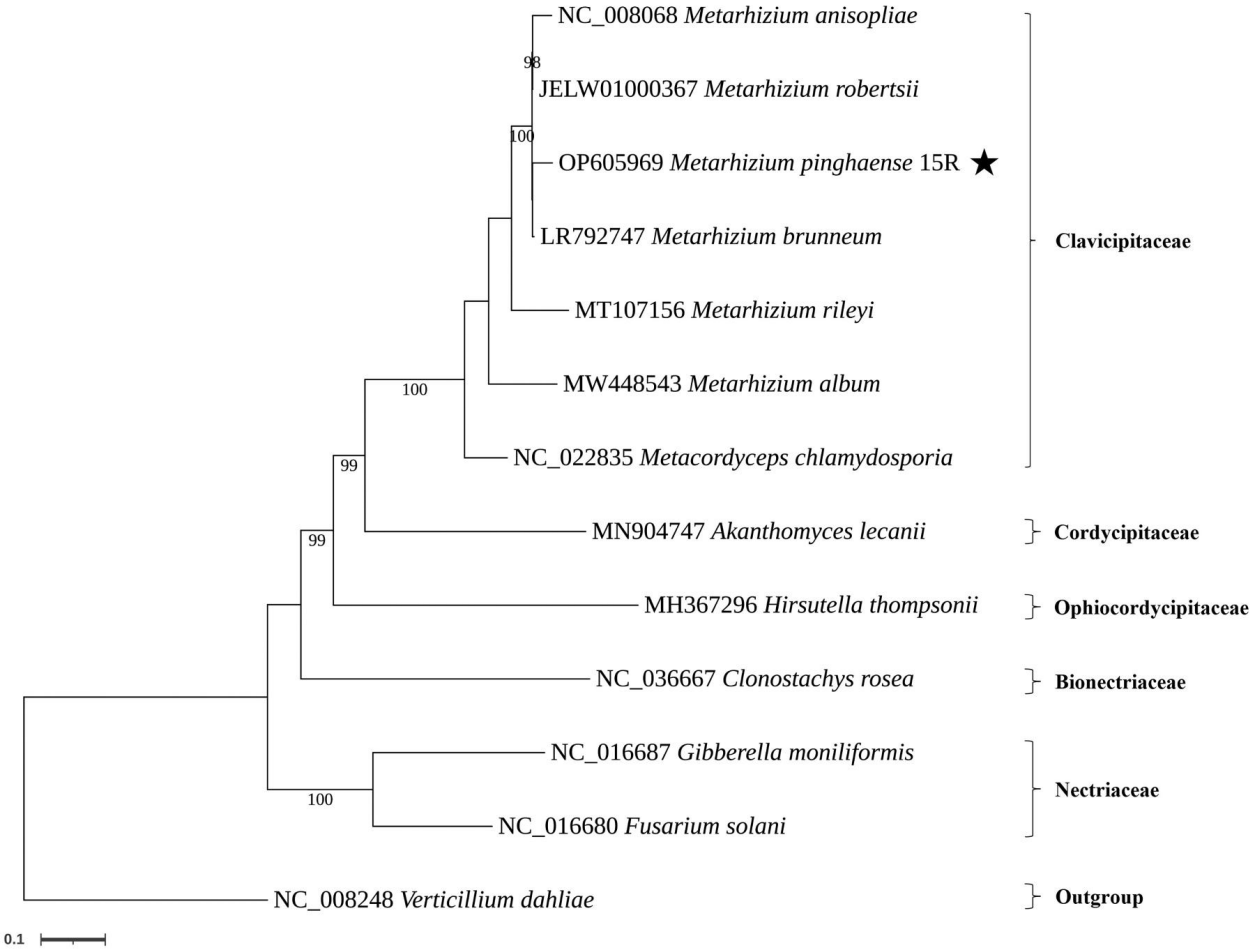
Phylogenetic trees based on the mitochondrial nucleotide sequence in related fungal species. Phylogenetic trees based on the mitochondrial nucleotide sequence in related fungal species. Phylogenetic tree of mitochondrial genomes of *Metarhizium pinghaense* 15 R and its related 12 species. We used seven species of Clavicipitaceae and representative species of other families with available mitogenomes in Hypocreales. Protein-coding sequences conserved in the mitochondrial genomes of 13 species were multiple-aligned using MAFFT (http://mafft.cbrc.jp/alignment/server/index.html) and used to generate a phylogenetic tree using the maximum likelihood approach as implemented in RAxML v8.2.12 (Stamatakis^2014^). the bootstrap values were indicated for nodes that support values of >70%. and *Verticillium dahliae* NC_008248.1 (Pantou et al. 2006) used an outgroup of this tree. The species under study is highlighted by the star. The following sequences were used: *Metarhizium album* MW448543.1 (Sun et al. 2021), *Metarhizium anisopliae* NC_008068.1 (Ghikas et al. 2006), *Metarhizium brunneum* LR792747.1 (Kortsinoglou et al. 2020), *Metarhizium rileyi* MT107156.1 (Zhang et al. 2020a), *Metarhizium robertsii* JELW01000367, *Akanthomyces lecanii* MN904747.1 (Zhang et al. 2020b), *Clonostachys rosea* NC_036667.1, *Fusarium solani* NC_016680.1 (Al-Reedy et al. 2012), *Gibberella moniliformis* NC_016687.1 (Al-Reedy et al. 2012), *Hirsutella thompsonii* MH367296.1 (Wang et al. 2018), *Metacordyceps chlamydosporia* NC_022835.1 (Lin et al. 2015), and *Verticillium dahliae* NC_008248.1 (Pantou et al. 2006).

The mitogenome of *M. pinghaense* 15 R (GenBank accession: OP605969.1) was a circular molecule of 35,763 bp with GC content of 27% (Figure 2). The average and maximum coverage were 3654.8x and 6445x, respectively (Figure S1). Identified genes include, the three ATP synthase subunits 6, 8, and 9 (*atp*6, *atp*8, and *atp*9), the three cytochrome oxidase subunits I, II, III (*cox*1, *cox*2, and *cox*3) and apocytochrome b (*cob*), the seven subunits of NADH dehydrogenase (*nad*1*, nad*2*, nad*3*, nad*4*, nad*4L*, nad*5, and *nad*6), the two ribosomal RNAs (*rnl* and *rns*), and 19 tRNA genes. Five genes were carrying a total of eight introns, including *cox*1*, cob*, and *rnl* with two introns while *cox*3 and *nad*1 with one intron each. These introns all belonged to the group I intron family, and they may encode ribosomal protein S3 (in *rnl*), LAGLIDADG (in *cob*, *cox*1, and *cox*3), and GIY-YIG endonucleases (in *cob*). The structures of the cis-splicing genes are shown in Figure S2. Phylogenetic analysis based on the mitochondrial nucleotide sequence (six conserved protein-coding sequences of *cob*, *cox*1, *cox*2, *nad*1, *nad*4, and *nad*5) by the Maximum Likelihood approach identified the *M. pinghaense* 15 R as a member of the Clavicipitaceae (Figure 3).

## Discussion and conclusion

This study was conducted as part of the work to characterize the *M. pinghaense* 15 R strain. The genus *Metarhizium*, the subject of this study, is one of the pathogenic fungi that cause insect diseases. Furthermore, these fungi have been produced and used as an environmentally friendly pest control method (Sullivan et al. 2022). At the same time as the preference for eco-friendly control measures is increasing, more microbial pesticides are being developed compared to the past, and research is also needed to secure the sovereignty of biological resources of strains developed as microbial pesticides (Lee et al. 2018). In our knowledge, current efforts to secure the sovereignty of these microorganisms include whole genome analysis, but there is no approach using mtDNA. Since it takes a lot of time and effort to analyze the whole genome (ca. 30 ∼ 41 Mb), which is relatively large in size, it is considered more accessible to secure the sovereignty of biological resources by securing the mtDNA sequence (ca. 24 ∼ 68 Kb). In other words, it is thought that the mtDNA sequence of the *M. pinghaense* 15 R strain reported in this study can be helpful as genetic data for biological resources.

So far, three species (*M. anisoplie* NC_008068.1, *M. brunnem* LR792747.1, and *M. robertsii* JELW01000367) of the *Metarhizium* have been analyzed for the main purpose of securing the mtDNA sequence, and only the mtDNA sequence of the other two species (*M. album* MW448543.1 and *M. rileyi* MT107156.1) has been determined through whole genome analysis. Of the approximately 55 species recorded in this genus, only 12% have been analyzed for mtDNA (Bischoff et al. 2009; Lopes et al. 2018; Mongkolsamrit et al. 2020;). This study is the first report analyzing and reporting the *Metarhizium pinghaense* mtDNA sequence to the best of our knowledge.

The number of tRNAs revealed the difference between *M. pinghaense* and *M. anisopliae*, despite their tight evolutionary relationship. In a previous study, *M. anisopliae* had 24 tRNAs (Ghikas et al. 2006), whereas *M. pinghaense* 15 R had 19 tRNAs. In comparing tRNA types, *M. pinghaense* 15 R have proline and valine, which were not confirmed in *M. anisopliae* of tRNA. In contrast, glutamine, phenylalanine, and threonine were not confirmed in *M. pinghaense* 15 R strain.

As a result, this fungus showed a close relationship with the existing *M. anisopliae* and *M. robertsii* of the same genus, which supports the recent differentiation of *M. anisopliae* and *M. guizhouense* (Mongkolsamrit et al. 2020). The *M. pinghaense* mtDNA sequence obtained through this study will be helpful for taxonomic and evolution studies as important data to which this fungus belongs.

## Supplementary Material

Supplemental Material

Supplemental Material

## Data Availability

The genome sequence data that support the findings of this study are openly available in GenBank of NCBI at https://www.ncbi.nlm.nih.gov/ under the accession no. OP605969. The associated BioProject, SRA, and Bio-Sample numbers are PRJNA815910, SRR18320042, and SAMN26650411, respectively.
