# Supplementary material for: The complete mitogenome of the entomopathogenic fungus Metarhizium pinghaense 15R
Source: Mitochondrial DNA B Resour. 2023 Dec 18;8(12):1411–5. doi: 10.1080/23802359.2023.2292145 (PMC10956925; doi:10.1080/23802359.2023.2292145)
Supplement: Supplemental Material [file TMDN_A_2292145_SM6775.docx]

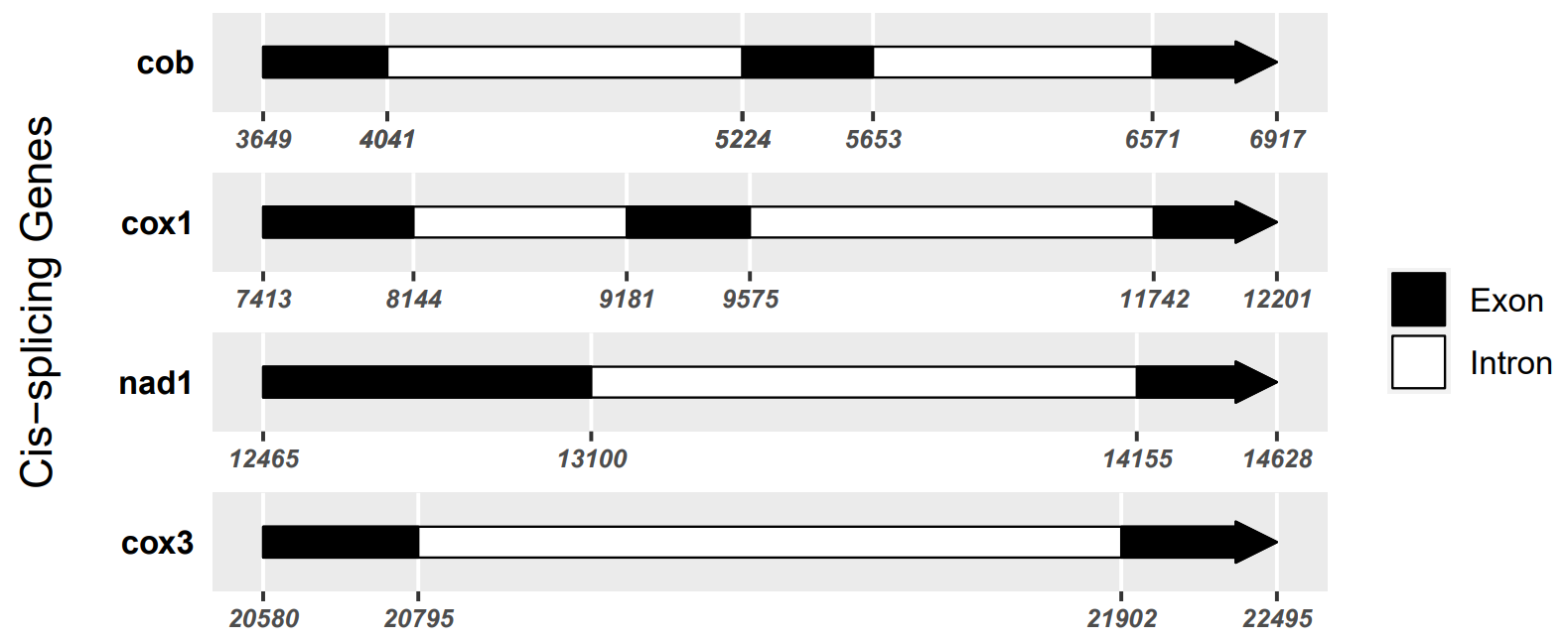


**Supplementary Figure S2**. **Schematic presentation of the structure of cis-splicing genes from the *Metarhizium pinghaense* 15R strain.** The white area is Intron, and the black area is the exon. The arrow shows the sense direction of the genes.
