# Supplementary material for: The complete mitogenome of the entomopathogenic fungus Metarhizium pinghaense 15R
Source: Mitochondrial DNA B Resour. 2023 Dec 18;8(12):1411–5. doi: 10.1080/23802359.2023.2292145 (PMC10956925; doi:10.1080/23802359.2023.2292145)
Supplement: Supplemental Material [file TMDN_A_2292145_SM6774.docx]

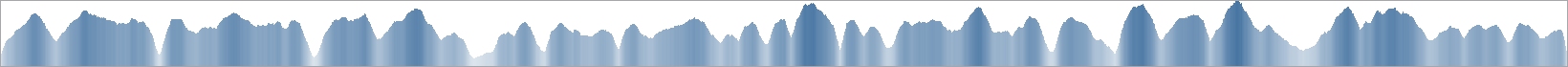


**Supplementary Figure S1. Read mapping depth of the plastome sequence**. Clean read mapping depth is presented with blue bars. X and Y axis present nucleotide position (1-35763 nt) and read mapping depth, respectively. The average and maximum coverage were 3654.8x and 6445x, respectively.
